# Very late extragonadal recurrence of testicular seminoma presenting as a bladder mass: a case report

**DOI:** 10.1097/MS9.0000000000005112

**Published:** 2026-05-07

**Authors:** Gassan Salih, Saad Masood, Fazeh Moafi, Chrysoula Fysaraki, Shabir Zarif

**Affiliations:** a Department of Urology, York and Scarborough Teaching Hospitals NHS Foundation Trust York, UK; b Department of Urology, York and Scarborough Teaching Hospitals NHS Foundation Trust York, UK; c Department of Urology, Balkh University, Mazar-i-Sharif, Balkh, Afghanistan

**Keywords:** case report, extragonadal recurrence, germ cell tumor, late relapse, testicular seminoma, urinary bladder metastasis

## Abstract

Very late relapse of testicular seminoma is rare and typically occurs at conventional metastatic sites such as the retroperitoneum, mediastinum, or lungs. Extragonadal recurrence involving the urinary bladder is particularly uncommon and may pose diagnostic challenges. We report the case of a 52-year-old male who presented with painless visible hematuria 16 years after undergoing radical inguinal orchidectomy for stage I testicular seminoma. Imaging revealed a large infiltrative mass arising from the left posterolateral wall of the urinary bladder, with extension into the prostate and distal ureter, resulting in severe hydronephrosis. Serum tumor markers, including alpha-fetoprotein, beta-human chorionic gonadotropin, and lactate dehydrogenase, remained within normal limits. Transurethral resection of the bladder tumor was performed, and histopathological examination, supported by immunohistochemistry, demonstrated features consistent with classical seminoma. The disease was characterized as locally advanced, with regional lymph node involvement and no evidence of distant metastases. Multidisciplinary management was initiated, including systemic platinum-based chemotherapy and urinary decompression. This case illustrates that seminoma may rarely recur after prolonged disease-free intervals and at atypical anatomical sites, with findings supporting late metastatic recurrence rather than a primary extragonadal germ cell tumor. It also emphasizes that normal tumor marker levels do not exclude recurrence and that histopathological confirmation remains essential for accurate diagnosis. Recognition of unusual presentations in long-term survivors of germ cell tumors is important to facilitate timely diagnosis and appropriate management.

## Introduction

Testicular seminoma is a highly curable germ cell tumor that accounts for approximately half of all testicular germ cell malignancies and primarily affects young and middle-aged men. Contemporary management with radical inguinal orchidectomy, followed by risk-adapted surveillance, chemotherapy, or radiotherapy, has resulted in excellent outcomes, with survival exceeding 95% in patients with early-stage disease^[^1^]^. Although prognosis is favorable, relapse can occur and most commonly develops within the first 5 years after treatment. Very late relapse, defined as recurrence occurring more than 10 years after apparent cure, is rare and represents an uncommon clinical scenario.

HIGHLIGHTS

- Very late relapse of testicular seminoma occurred 16 years after the initial treatment.
- Recurrence presented as a muscle-invasive bladder mass, mimicking urothelial carcinoma.
- Serum tumor markers remained normal despite significant local disease.
- Histopathology and immunohistochemistry confirmed seminoma recurrence.
- Long-term vigilance is important in survivors of testicular germ cell tumors.

When recurrence occurs, the disease most frequently involves the retroperitoneal lymph nodes, mediastinum, or lungs. In contrast, extragonadal relapse at atypical anatomical sites is uncommon^[^2^]^. Extragonadal germ cell tumors account for a small proportion of cases and are typically located along the midline, most commonly in the retroperitoneum or mediastinum^[^2^]^. Involvement of the urinary bladder by seminoma is particularly unusual and may mimic primary urothelial carcinoma both clinically and radiologically. This overlap can delay diagnosis, especially because serum tumor markers may remain within normal limits in patients with pure seminoma despite significant tumor burden.

In such situations, histopathological examination supported by immunohistochemistry is essential for establishing an accurate diagnosis. Recognition of these rare presentations is important because seminoma remains highly responsive to platinum-based chemotherapy, even in recurrent disease, and early identification may influence treatment strategy and prognosis^[^3^]^. Furthermore, distinguishing metastatic recurrence from a primary extragonadal germ cell tumor is clinically important, as this distinction has implications for disease interpretation and management. Transparent reporting of rare clinical presentations is therefore valuable for expanding the clinical literature. This case report was prepared in accordance with the established case-reporting recommendations and the TITAN reporting framework for transparency in manuscript preparation^[^4^]^. No artificial intelligence tools were used in the writing or generation of this manuscript.

We report a case of very late recurrence of testicular seminoma presenting at an atypical extragonadal site as a muscle-invasive bladder mass 16 years after initial treatment. This report highlights the diagnostic challenges associated with atypical metastatic sites and normal tumor marker profiles, and emphasizes the importance of careful evaluation of new urological symptoms in long-term survivors of germ cell tumors.

## Case presentation

A 52-year-old male was referred to a tertiary care center in October 2025 with a several-week history of painless visible hematuria accompanied by intermittent left-sided flank discomfort. He denied fever, weight loss, dysuria, or lower urinary tract symptoms. Initial assessment at the referring institution demonstrated left-sided hydronephrosis and a bladder mass measuring approximately 5 cm on cystoscopic examination.

The patient had a significant oncological history of left testicular seminoma, diagnosed in 2009, which had been treated with radical inguinal orchidectomy. At the time of the current presentation, he reported no other major comorbidities and was not receiving ongoing oncological treatment.

On physical examination, the patient was hemodynamically stable and afebrile. Abdominal examination revealed no palpable masses or organomegaly. Digital rectal examination demonstrated an indurated area involving the left lobe of the prostate, raising concern for locally invasive disease. Baseline laboratory investigations at presentation are summarized in Table 1 and demonstrated normal tumor marker levels with preserved function of the contralateral kidney.

**Table 1:** Baseline laboratory investigations at presentation.

Parameter	Result	Reference range	Clinical interpretation
Hemoglobin	12.8 g/dL	13–17 g/dL	Mild anemia consistent with chronic blood loss
Total leukocyte count	7.6 × 10^9^/L	4–11 × 10^9^/L	Within normal limits
Platelet count	312 × 10^9^/L	150–400 × 10^9^/L	Adequate marrow reserve
Serum creatinine	1.5 mg/dL	0.6–1.3 mg/dL	Mild renal impairment likely secondary to obstructive uropathy
Blood urea nitrogen	28 mg/dL	7–20 mg/dL	Elevated, consistent with obstructive renal dysfunction
Serum sodium	138 mmol/L	135–145 mmol/L	Within normal limits
Serum potassium	4.4 mmol/L	3.5–5.0 mmol/L	Within normal limits
Alanine aminotransferase	32 U/L	<40 U/L	Within normal limits
Aspartate aminotransferase	29 U/L	<40 U/L	Within normal limits
Alkaline phosphatase	118 U/L	40–129 U/L	No biochemical evidence of hepatic or osseous involvement
Lactate dehydrogenase	210 U/L	140–280 U/L	Within normal limits
Alpha-fetoprotein	3.4 ng/mL	<10 ng/mL	Normal; not suggestive of nonseminomatous germ cell tumor
Beta-human chorionic gonadotropin	2.1 IU/L	<5 IU/L	Within normal limits
C-reactive protein	9 mg/L	<5 mg/L	Mild inflammatory response
Urinalysis	Gross hematuria; no casts	Not applicable	Consistent with tumor-related bleeding

AFP, alpha-fetoprotein; ALT, alanine aminotransferase; AST, aspartate aminotransferase; CRP, C-reactive protein; LDH, lactate dehydrogenase; β-hCG, beta-human chorionic gonadotropin

**Figure 1.**
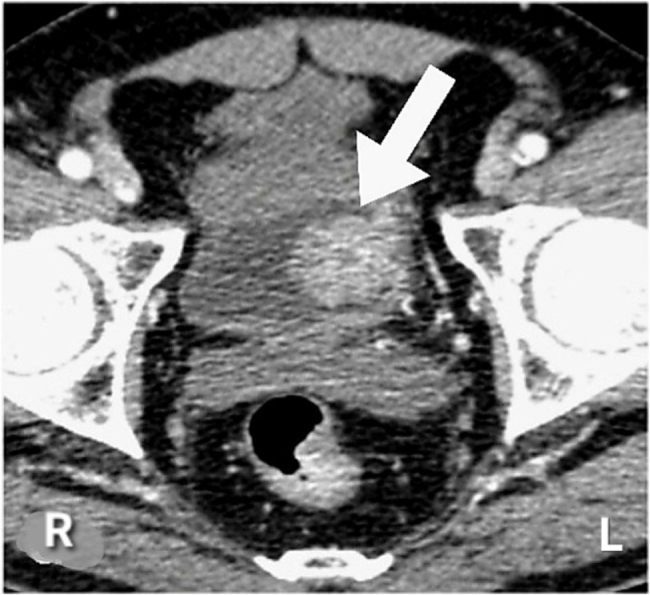
Contrast-enhanced computed tomography urogram demonstrating a heterogeneous soft-tissue mass arising from the left posterolateral wall of the urinary bladder with intraluminal extension (arrow). The lesion demonstrates local invasion toward adjacent pelvic structures, consistent with a locally advanced bladder mass. CT, computed tomography.

A contrast-enhanced computed tomography urogram revealed a large, irregular, infiltrative mass measuring approximately 8 cm, arising from the left posterolateral wall of the urinary bladder. The lesion demonstrated heterogeneous enhancement with intraluminal extension, as illustrated in Fig. 1. The mass extended contiguously into the left lobe of the prostate and the distal left ureter, resulting in complete ureteric obstruction. Local extension toward the left pelvic sidewall was also observed.

Marked left-sided hydronephrosis with severe cortical thinning of the kidney was present, consistent with chronic obstructive uropathy. Multiple enlarged left external iliac lymph nodes were identified, producing mild indentation of adjacent vascular structures without evidence of venous obstruction. No radiological evidence of distant metastatic disease or aggressive osseous lesions was identified. The right kidney appeared normal, except for a small, simple cortical cyst.

The patient subsequently underwent transurethral resection of the bladder tumor (TURBT) for diagnostic confirmation and staging. Intraoperatively, a large, solid, infiltrative tumor was identified arising from the left posterolateral bladder wall, with clear evidence of muscle invasion.

Histopathological examination of the resected tissue demonstrated extensive infiltration of the bladder wall by a malignant neoplasm composed of large, uniform cells arranged in sheets and lobules, separated by delicate fibrous septae containing prominent vascular channels. The tumor cells exhibited abundant clear to pale cytoplasm with distinct cell borders, centrally located polygonal nuclei, finely granular chromatin, and one to two prominent nucleoli. A dense lymphocytic infiltrate was present within the fibrous septae, accompanied by a focal granulomatous reaction. The tumor infiltrated the urothelium and extended deeply into the detrusor muscle. Microscopic examination demonstrated features characteristic of classical seminoma, including sheets of large polygonal cells with clear, glycogen-rich cytoplasm and fibrous septa containing lymphoid infiltrates.

Immunohistochemical analysis was performed to further characterize the tumor and exclude other potential malignancies. The neoplastic cells demonstrated strong nuclear positivity for OCT3/4, with diffuse membranous and cytoplasmic expression of placental alkaline phosphatase (PLAP) and CD117. Focal positivity for cytokeratin AE1/AE3 was also observed. In contrast, the tumor cells were negative for CD30, GATA3, CK7, chromogranin, synaptophysin, NKX3.1, and CD45. The immunohistochemical findings and their diagnostic significance are summarized in Table 2. In the context of the patient’s previous history of testicular seminoma and the characteristic histomorphological and immunophenotypic profile, these findings confirmed classical seminoma involving the urinary bladder, consistent with a very late extragonadal recurrence.

**Table 2:** Immunohistochemical markers and diagnostic interpretation.

Marker	Result	Diagnostic interpretation
OCT3/4	Strong nuclear positivity	Supports diagnosis of seminoma
PLAP	Diffuse membranous and cytoplasmic positivity	Germ cell tumor marker consistent with seminoma
CD117 (c-KIT)	Positive	Typical immunophenotypic marker of classical seminoma
Cytokeratin AE1/AE3	Focal positivity	Occasional epithelial marker expression in seminoma
CD30	Negative	Helps exclude embryonal carcinoma
GATA3	Negative	Helps exclude urothelial carcinoma
CK7	Negative	Not consistent with urothelial carcinoma
Chromogranin	Negative	Excludes neuroendocrine tumor
Synaptophysin	Negative	Excludes neuroendocrine tumor
NKX3.1	Negative	Excludes prostatic adenocarcinoma
CD45	Negative	Excludes lymphoma

PLAP, placental alkaline phosphatase

Review of the patient’s medical records confirmed that he had undergone a left transinguinal radical orchidectomy in 2009, following presentation with a painless left testicular mass. Preoperative scrotal ultrasonography demonstrated a solid intratesticular lesion, while serum tumor markers were within normal limits. Histopathological evaluation of the orchiectomy specimen revealed classical seminoma confined to the testis and staged as pT1NxMx according to the tumor–node–metastasis (TNM) classification. A postoperative staging CT scan showed no evidence of metastatic disease.

The patient was subsequently managed with active surveillance at a tertiary oncology center. Follow-up included regular clinical assessments, serial tumor marker measurements, and periodic imaging over 5 years, all of which demonstrated no evidence of recurrence. In 2014, a left testicular prosthesis was inserted, and he was discharged from routine oncological follow-up later that year in complete remission.

Based on the current radiological findings, histopathological confirmation, and regional nodal involvement, the disease demonstrated locally advanced bladder involvement with regional lymph node metastases. Although the extent of local invasion was initially described using bladder cancer TNM terminology, this was applied descriptively to convey anatomical extent and does not represent formal staging for seminoma. The case was reviewed at the institutional germ cell multidisciplinary team meeting because of the unusual site of recurrence and locally advanced presentation. Given the severe left-sided hydronephrosis with marked cortical thinning, urgent urinary decompression using ureteric stenting or percutaneous nephrostomy was recommended to preserve renal function.

Systemic platinum-based chemotherapy with a bleomycin, etoposide, and cisplatin (BEP) regimen was recommended as first-line therapy due to the known chemosensitivity of seminoma in recurrent disease. The potential role of consolidative surgery, including radical cystectomy with pelvic lymphadenectomy, was reserved for reassessment after the completion of chemotherapy, depending on the treatment response. Radiotherapy was also considered as a potential adjunct for locoregional control if required. Given the normal tumor marker profile and stable clinical status, a structured plan for close radiological and biochemical surveillance was established.

## Discussion

Late relapse of testicular seminoma is uncommon, and recurrence occurring more than 10 years after initial remission is rare. Most relapses develop within the first 5 years after treatment and typically involve the retroperitoneum, mediastinum, or lungs. Extragonadal recurrence at atypical anatomical sites is unusual, and involvement of the urinary bladder has only rarely been described^[^5^]^. Only a limited number of cases describing bladder involvement by seminoma have been reported in the literature, most often in the context of advanced or disseminated disease, further underscoring the rarity of this presentation. The present case is notable for the prolonged disease-free interval of 16 years and for the uncommon site of recurrence with locally invasive behavior. Such presentations highlight the possibility of prolonged tumor dormancy and the variable long-term biological behavior of seminoma.

The mechanisms underlying late and extragonadal relapse remain incompletely understood. Proposed explanations include reactivation of dormant micrometastatic disease, delayed progression of previously undetected microscopic deposits, or malignant transformation of residual ectopic germ cells.^[^6^]^ The urinary bladder is not a typical site for germ cell tumor metastasis, making this presentation particularly unusual. An additional diagnostic consideration in such cases is the distinction between late metastatic recurrence and a primary extragonadal germ cell tumor arising *de novo*. Primary extragonadal germ cell tumors are rare and typically arise along midline structures, such as the mediastinum or retroperitoneum, with bladder involvement being exceedingly uncommon. In the present case, the patient’s prior history of classical testicular seminoma, the concordant histomorphological and immunophenotypic findings, and the pattern of regional nodal involvement collectively favor a diagnosis of late metastatic recurrence rather than a *de novo* extragonadal tumor. In addition, the extensive invasion into adjacent pelvic structures observed in this patient illustrates how late recurrent seminoma may mimic aggressive primary pelvic malignancies both clinically and radiologically, potentially leading to diagnostic uncertainty.

This case also highlights the limitations of serum tumor markers in detecting recurrent seminoma. Alpha-fetoprotein is generally normal in pure seminoma, and both beta-human chorionic gonadotropin and lactate dehydrogenase may remain within reference ranges despite significant tumor burden.^[^7^]^ In the present case, tumor marker levels were normal despite extensive local disease and regional nodal involvement. These findings emphasize that normal tumor markers do not exclude recurrence and that new urological or obstructive symptoms in long-term cancer survivors warrant thorough radiological and histopathological evaluation.

Histopathological examination, supported by immunohistochemistry, was essential for establishing the diagnosis. The characteristic morphology, together with strong expression of OCT3/4, placental alkaline phosphatase, and CD117, confirmed classical seminoma while excluding other potential diagnoses such as urothelial carcinoma, lymphoma, neuroendocrine tumors, and prostate adenocarcinoma.^[^8^]^ The differential diagnosis in this context is broad and includes primary urothelial carcinoma, lymphoma, neuroendocrine tumors, and prostatic adenocarcinoma, all of which were effectively excluded based on the immunohistochemical profile. Accurate pathological classification is important because management strategies differ substantially among these conditions. Seminoma remains highly responsive to platinum-based chemotherapy even in recurrent disease, and early recognition therefore allows the possibility of effective treatment. Multidisciplinary management is recommended, with systemic chemotherapy forming the mainstay of therapy and additional surgical or radiotherapeutic interventions considered according to treatment response.^[^9^]^ Although the anatomical extent of disease in this case was described using bladder cancer TNM terminology for clarity, it is important to emphasize that germ cell tumors follow distinct staging systems, and such terminology was applied descriptively rather than for formal staging purposes.

This case also has implications for long-term follow-up in patients with testicular seminoma. Current surveillance protocols for early-stage disease frequently limit routine follow-up to approximately 5 years after treatment. However, rare instances of very late relapse may occur and can present with locally advanced disease. Continued clinical awareness and patient education regarding new or unexplained urological symptoms may, therefore, facilitate earlier recognition of recurrence in long-term survivors of germ cell tumors.

## Conclusion

This case describes a rare instance of a very late extragonadal recurrence of testicular seminoma presenting as a muscle-invasive bladder mass 16 years after the initial treatment. The case illustrates that normal tumor marker levels do not reliably exclude recurrence and that atypical metastatic patterns may closely resemble primary urological malignancies, potentially leading to diagnostic delay. Careful evaluation of new urological symptoms in patients with a remote history of germ cell tumors is therefore essential. Histopathological confirmation with immunohistochemistry remains critical for establishing the correct diagnosis. Despite locally advanced presentation, seminoma retains substantial chemosensitivity, highlighting the importance of timely multidisciplinary management with curative intent even in very late relapse.

## Data Availability

The data supporting the findings of this case report are contained within the article. Additional clinical information cannot be publicly shared in order to protect patient confidentiality but may be made available from the corresponding author upon reasonable request and with appropriate ethical considerations.
